# Novel analysis tool for the distance of gold dimers controlled by the DNA strand length on the DNA origami

**DOI:** 10.1111/jmi.13371

**Published:** 2024-11-15

**Authors:** Jannik Guckel, Zhe Liu, Zunhao Wang, Birka Lalkens, Markus Etzkorn, Daesung Park

**Affiliations:** ^1^ Physikalisch‐Technische Bundesanstalt Braunschweig Germany; ^2^ Laboratory for Emerging Nanometrology (LENA) Braunschweig Germany; ^3^ Institute of Applied Physics Technische Universität Braunschweig Braunschweig Germany; ^4^ Institute of Semiconductor Technology Technische Universität Braunschweig Braunschweig Germany

**Keywords:** circle Hough transform, DNA origami, gold nanoparticles, interparticle distance

## Abstract

Metallic nanoparticle dimers have been used to enhance the excitation rate of single‐quantum emitters. The interparticle distance (*d*) of the dimers has a crucial influence on the signal enhancement. Therefore, precise control of *d* is desired for optimal performance. However, statistical analysis of *d* has been often restricted to a small number of dimers due to the lack of reliable automatic software tools.

For this reason, we developed a novel analysis tool for automatic dimer analysis. Our approach combines particle detection by circle Hough transformation (CHT) with custom classification routines optimised for distinct types of particles. We applied our tool to scanning electron microscopy (SEM) images and achieved great agreement in dimer detection, reaching an agreement of around 97% between automatic analysis and manual inspection for more than 3000 metallic nanoparticle dimers on DNA origami controlled by a combination of multiple DNA strands.

Our study revealed the effects of the strand length (*L*) on the distribution of *d*. Based on geometric consideration, we expected a strong correlation between *L* and the standard deviation (*σ*) of *d*. We could verify this correlation by characterising four dimer designs with different *L* while analysing more than 1000 dimers per specimen.

## LAY DESCRIPTION

Metallic nanoparticle pairs, known as dimers, have been used as antennae for single‐quantum emitters. The distance (*d*) between the dimer particles has a crucial influence on its signal enhancement. Therefore, precise control of *d* is desired for optimal performance. However, investigating variations in *d* between nominally identical dimers is laborious due to the lack of reliable automatic software tools.

Therefore, we developed a novel analysis tool for automatic dimer analysis in secondary electron microscopy (SEM) images. We use the circle Hough transform (CHT) to detect the spherical particles and novel analysis routines for the identification of dimers and other distinct types of particles. We applied our tool to a set of SEM images, which feature different specimens of dimers on DNA origami. We achieved an agreement of 97% of automatically analysed dimers (more than 3000 dimers), which were manually inspected.

Additionally, our study investigates the differences between the dimer specimens. Our dimers were assembled on the DNA origami by using DNA strands. The length (*L*) of the DNA strands was changed between the specimens. Based on geometric consideration, we expected a strong correlation between strand length *L* and the standard deviation (*σ*) in the distribution of *d*. We could verify this correlation by characterising four specimens with different *L*, while analysing more than 1000 dimers per sample.

## 1 INTRODUCTION

Gold nanoparticles have a wide range of applications due to their beneficial properties, such as being biocompatible, chemically inert, easily customisable, and having good catalytic properties.^1^, ^2^, ^3^ Therefore, they became attractive to biomedical applications such as drug delivery or nanozymes.^4^, ^5^

Furthermore, they are often used for signal enhancement in surface‐enhanced Raman spectroscopy (SERS). The intensity of the Raman signal is increased by several orders of magnitude if molecules are located close to a rough noble‐metal surface. Visible light excites the local surface plasmon resonances of the metal, leading to strong electromagnetic fields near sharp surface features, which in turn increase the polarisation of nearby molecules.^6^ In addition, the surface features also absorb the signal emitted at the near‐field by these molecules and radiate it to the far‐field, thus finally making it act as an antenna in the nanoscale.^7^ Moreover, the ensembles of nanoparticles also can enhance the additional electromagnetic fields in the gaps between particles.^8^ Several experiments showed that this additional signal enhancement increases with reduced gap size.^9^, ^10^, ^11^

This signal enhancement resulting from the collective effects of nanoparticles can be expected in the photon emission of single‐quantum emitters. The presence of coupled nanoparticles (dimers) was shown to increase the fluorescence by a factor of 30.^12^ Palomba et al.^13^ showed that the resonance frequencies of such antennae depend on the interparticle distance (*d*).

For these reasons, precisely controlling the *d* of the dimers becomes an important aspect when the dimers are used for quantum emitters. A straightforward method to obtain information about *d* is to take images of dimers using an electron microscope with the benefit of superior spatial resolution and analyse *d* between dimers from those images. However, it becomes difficult to accurately measure *d* from a large number of microscopy images containing numerous dimers located in complex systems. Manual analysis is a laborious and time‐consuming task. An automatic analysis requires reliable solutions for the identification of dimers, their precise positions, and robust distance measurement. Despite such a solution being desired, the automatic analysis of a large number of dimers from electron microscopy images has not been easily available. Therefore, providing results of statistical analysis for *d* is restricted by a small number of images of dimers.^14^ To overcome this issue, we propose a novel analysis tool for the automatic dimer identification and measurement of interparticle distance in this study.

We applied our tool to a material system with gold dimers controlled by a DNA origami system. We achieved precise position control of the gold dimers by connecting the nanoparticles onto the origami with single DNA strands out of poly‐thymine and poly‐adenine. In addition, the design of the dimer avoids a direct connection between dimer particles, for example, by using linker molecules.^12^ Without a rigid connection between them, significant variations of *d* are expected. Therefore, a small sampling size of the given complex system would not be sufficient, leading to a statistically inaccurate analysis of *d*. For this material system, approximately 1000 dimers were required for each configuration to accomplish a reliable analysis of the expected effects of the material parameters on the distribution of *d*. Therefore, a reliable automatic analysis tool is essential to obtain high‐efficiency analysis results.

## 2 MATERIALS AND METHODS

### 2.1 Modelling the distribution of *d*

In this section, we describe a simple model for the estimation of plausible interparticle distances in the target system. We define *d* as the centre‐to‐centre distance between the nanoparticles from a top‐down view.

**FIGURE 1:**
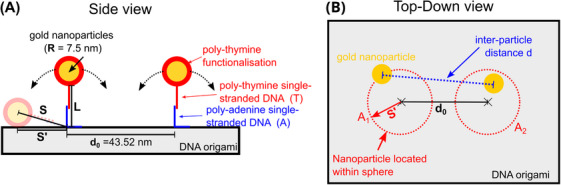
Geometric model of the distance distribution: (A) side view of the suggested model showing projection distance, and (B) top‐down view of our model. We assume the nanoparticles to drop within the circles *A*_1, 2_ of radius *S*’ around the anchor points.

Both gold nanoparticles are connected to the DNA origami by a DNA strand with the strand length (*L*) (Figure 1A). We assumed the particle radius of *R* = 7.5 nm based on the nominal diameter provided by the supplier. The DNA strand consists of poly‐thymine (T) and poly‐adenine (A) single‐stranded DNA with a segment of double‐stranded DNA connecting them. The end of each adenine strand is anchored to the DNA origami, and the end of the thymine directly connects to the particle. The gap between both anchor points (*d*_0_) is fixed at 43.52 nm by the design of the DNA origami.

The straight‐line distance *S* between an anchor point and the centre of the nanoparticle is assumed as

(1) S=L+R.

The maximum distance in the top‐down view between the centre of a nanoparticle and the anchor point is calculated by the projection of *S* onto the surface of the DNA origami given by

(2) $$S^{\prime }=\sqrt{S^{2}-R^{2}.}\;$$

In our model, the position of the nanoparticles is not fixed due to the flexibility of *L*. Figure 1B shows our model in the top‐down view. The cross marks on the DNA origami are the anchor points in Figure 1A. Each nanoparticle can be located within the red circle with radius *S'* (*A*_1_, *A*_2_) around its anchor point.

We assumed an even distribution for the particle position within each circle. This was realised by using a circular Fibonacci grid with 500 points for each circle. We estimated the distribution of *d* by calculating the distances of all 250,000 possible connections between the points on *A*_1_ with *A*_2_.

Based on our geometric model, the length of the connecting DNA strand is expected to have a crucial influence on the standard deviation (*σ*). Longer DNA strands allow the particle to move farther away from its anchor point, expecting an increase in the *σ* of *d*. Therefore, we investigated four specimens with different *L*. Table 1 provides the detailed information about the specimens examined. The specimens are named after the configuration of their DNA strands, with the letters T and A representing thymine and adenine, respectively. The numbers following each letter indicate the number of base pairs for each amino acid. The *L* for each specimen was calculated using the model presented in Supplementary Section S2. *S’* was calculated based on Equations (1) and (2).

**TABLE 1:** Summary of strand lengths for the different specimens.

Specimen name	*L* (nm)	*S*’ (nm)
T12A9	5.09	10.11
T12A12	5.6	10.74
T19A12	6.81	12.18
T19A25	8.77	14.43

### 2.2 Synthesis of self‐assembled dimers with variations in *L*

Our synthesis method was adapted from the method suggested by Gür et al.^15^ and improved by including minor modifications. We used this method to synthesise four specimens with different *L* by varying the length of the poly‐adenine and thymine strands. More details on our synthesis procedure can be found in the Supplementary Section S1. The synthesis of the dimers consists of three steps: in the first step, we treated gold nanoparticles with nominal diameter of 15 nm from the BBI solutions in a solution of bis(p‐sulfonatophenyl) phenylphosphine dihydrate dipotassium salt (BSPP) and functionalised the particle surface with single‐stranded poly‐thymine DNA. In the second step, the DNA origami structures are modified with each DNA origami having the 6 staples extended by poly‐adenine single‐stranded DNA (three at each anchor point). In the final step, the modified DNA origami structures are mixed with the gold nanoparticles at a molar ratio of 1:50. The functional groups of both DNA strands (poly‐thymine and adenine) are prone to connect each other and create a double‐stranded DNA segment. The incubation period for this process lasted for 90 min. The mixture is then purified by gel electrophoresis, using a mixture of an agarose gel with a buffer (0.75 g gel per 100 mL buffer). The buffer is a tris acetate EDTA buffer (TAE) and contains 11 mM magnesium chloride (MgCl_2_). The fraction of gel containing the gold dimers is cut. The dimer‐containing liquid is squeezed out and collected between two glass slides.

### 2.3 Specimen preparation and image acquisition

The solution containing the dimers was deposited on an oxygen‐plasma treated silicon substrate using the drop‐casting method. The silicon substrate was first exposed to an oxygen plasma for 20 s for the creation of hydrophilic silanol on the surface to increase the rate of dimer adsorption.^16^ A 2 µL drop of the synthesised solution was placed on the silicon surface. Subsequently, the specimen was hermetically sealed in a petri dish and incubated in a humid atmosphere for 1 h. The substrate was washed for 5 min with an automated shaker in a buffer (40 mM Mg^2+^, 40 mM tris, solved in milliQ water, pH 8.3) and cleaned by submerging the substrate into three ethanol‐water mixtures with different volume fractions of ethanol (50%, 75%, 85%). The substrate was exposed to the 50% mixture for 10 s, followed by the 75% mixture for 20 s and the 85% mixture for 2 min. As a last step, the specimen is dried.

A Thermo Fisher Scientific Helios 5 UX dual beam FIB‐SEM was used to characterise the prepared specimens. The SEM images were acquired at an acceleration voltage of 2 kV using a through‐the‐lens detector (TLD) to reduce the shadow effects caused by the equipped side detector. We used 3072 × 2188 pixels per image (0.697 nm/pixel). Low beam currents, ranging from 13 to 50 pA, were used to reduce electron beam‐induced charging and contamination effects. These imaging conditions were suitable for achieving high contrast between the particles, the origami, and the substrate.

### 2.4 Automatic analysis of dimer statistic

#### 2.4.1 Visible particle classes

The main feature of our software is the automatic identification and classification of dimers within the DNA origami. For this purpose, it is necessary to image all crucial objects, including DNA origami, nanoparticles, and the substrate with SE. However, the connecting DNA strands cannot be directly imaged using SEM owing to their low contrast. To resolve this issue, we utilised robust criteria to define a dimer in this system.

**FIGURE 2:**
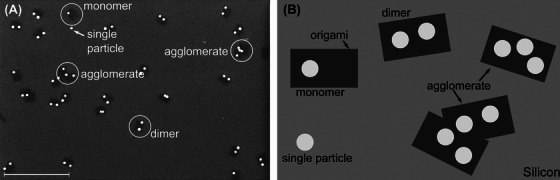
Particle classification of the different structures observed in top‐down view: (A) SE image and (B) simplified sketch. The scale bar in (A) represents 500 nm.

Qualitative image analysis by hand reveals four common structures, for which a set of quantitative and qualitative criteria can be defined (Figure 2). The most important structure is the dimer. We assume that the dimers are a pair of nanoparticles on the same origami as *d < d*_max_, with *d*_max_ as the maximum value of *d* based on our model.

Furthermore, we classified monomers, single particles, and agglomerates. A monomer is defined as a single nanoparticle within an origami, whereas single particles are defined as the particles located directly on the wafer. Finally, we define the agglomerates as groups of particles, in which the particles can theoretically be classified as either monomer or dimer. The classification ambiguity is created by their proximity to each other. Therefore, some particles have more than one plausible particle to form a dimer with. These particles cannot be classified with the information provided by the image. For this reason, we define an additional class for these particles. In addition to that, the particles in the proximity of the ambiguous particles are also included as part of the agglomerate because their options for dimer pairs include ambiguous particles. Therefore, the choice of particle class depends on particles which cannot be classified. The simplest case of agglomeration is achieved with more than 2 particles on the same origami. Furthermore, complex origami overlap can lead to agglomeration.

The classification criteria of our software are inspired by the criteria used in the manual analysis of this complex system covering extensive possibilities of variations.

### 2.5 Detection of spherical nanoparticles

**FIGURE 3:**
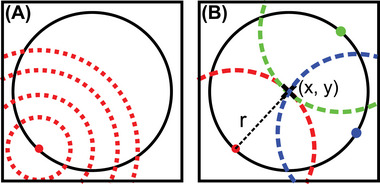
Detection approach of the circle Hough transform: (A) an edge pixel is selected, and potential circles are drawn. This step is executed for every edge pixel and (B) for a perfect circle with a known radius, 3 edge pixels are sufficient to find the centre position.

We utilised the circle Hough transform (CHT) to detect the spherical particles in our images. The CHT is an efficient method to detect spherical objects by analysing the contours of all features in digital images. The CHT detection approach is schematically explained in Figure 3.

In the first step, the edges of the particles are detected by canny edge. In the next step, each edge pixel is used as a circle centre and all possible circles within a defined radius interval are drawn (Figure 3A). Then, the CHT algorithm checks each image pixel (*x*, *y*) and counts the circles with radius r that intersect it (Figure 3B). A score, that is equal to the number of intersecting circles, is assigned to all combinations of (*x*, *y*, *r*). A positive detection result corresponds to a local score maximum in this matrix.

Finding the local maximum can be alternatively understood as a general mathematical expression to determine the centre of a perfect circle with a known radius (Figure 3B). In this specific case, determining the intersection from the 3 points on the contour is sufficient to find the centre of the circle. However, the radius can only be estimated in practice and a real nanoparticle does not have a perfectly spherical shape. These issues can be resolved by applying the CHT algorithm, since it evaluates every pixel on the edges with a combination of a radius variation. The maximum local score corresponds to the fit with the highest agreement based on the detected contour. Thus, our software uses the CHT algorithm implemented in the OpenCV library to generally detect the spherical nanoparticles.^17^

### 2.6 Automatic classification of nanoparticles

As shown in Figure 2, a robust automatic detection of the dimers located within the DNA origami requires information about the nanoparticles and the DNA origami. For this purpose, we must first identify the DNA origami and nanoparticles on each origami before the classification.

**FIGURE 4:**
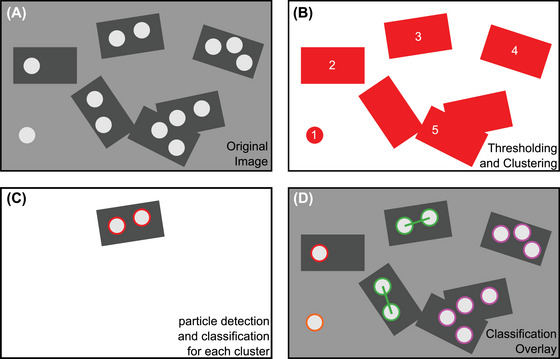
Workflow of the automatic analysis: (A) the original image containing particles, origami, and wafer, (B) the image after thresholding and labelling identifies structures containing particles as pixel clusters, (C) individual analysis of a pixel cluster by CHT. The particles are classified before moving to the next cluster, and (D) after classification, the particles are highlighted in the original image for visual feedback. Separate colours are used for each particle class. Orange is used for single particles without an underlying origami, red is used for monomers, green is used for dimers and purple is used for agglomerates.

Our workflow is shown in Figure 4, explaining how we analyse each image and classify the particles.

**FIGURE 5:**
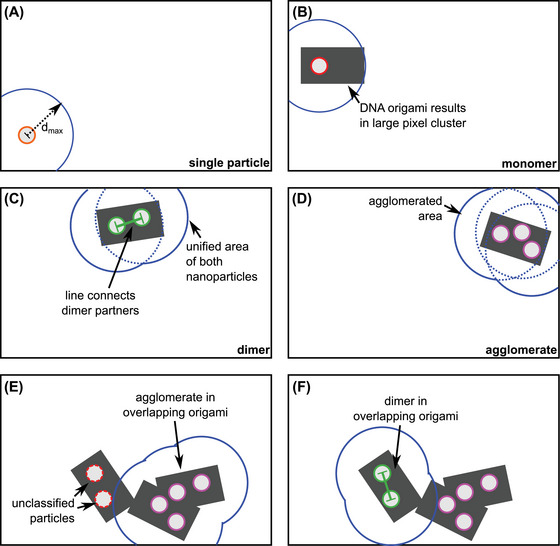
Automatic particle classification algorithm for complex pixel clusters. (A)–(D) represent one of the four particle classes in the algorithm: (A) single particle without DNA origami, (B) monomers, (C) dimers, and (D) agglomerates. Different colours are used to quickly identify each particle class in the final analysis. (E) and (F) represent a complex origami overlap. The algorithm checks for particles of each class independently. Therefore, an agglomerate and a dimer can exist on the same pixel cluster.

We apply intensity thresholding to eliminate the wafer area from the rest of the image. In the next step, we assign each pixel cluster an individual label for the cluster analysis (Figure 4B). Next, the algorithm selects a single pixel cluster by applying a robust mask function and applies the CHT algorithm to this image (Figure 4C). We developed a novel algorithm to determine the class of each particle. The algorithm developed is explained in Figure 5. The last two steps are repeated for all kinds of pixel clusters. To ensure the detection quality to the user, the algorithm provides an image with the detected particles highlighted in different colours (Figure 4D). Each colour stands for a separate particle class. We use this image as visual check to identify poorly detected dimers, which were manually removed for further analysis.

The goal is to classify every particle into one of the four classes introduced in Figure 2. The class of each particle is decided by the distances between different particles in the same pixel cluster, which leads to unique criteria for each class. The algorithm examines a certain area around each nanoparticle, which is symbolised by the solid blue circle around the centre of the particle in Figure 5A. Its radius is set to be identical to the *d*_max_ between the dimer particles. Therefore, the other particles inside this area are considered as potential second particles of a dimer.

If no other particle is found within *d*_max_, the nanoparticle can be classified as a single particle or a monomer. To distinguish a single particle and a monomer, the total area of the pixel cluster is measured, as the area of DNA origami is much larger than a single nanoparticle (Figure 5A and B).

The algorithm defines a dimer as two particles, which only have each other as a dimer option. Therefore, only the second particle can be found within *d*_max_ of the first particle, and vice versa for the second particle. These conditions can alternatively be checked by examining the unified area around both particles, marked by a solid blue line in Figure 5C. The area around each particle is represented by the dotted blue lines and is defined in analogy to Figure 5A. If exactly two particles are found within the unified area, the pair is considered a dimer. In addition, the interparticle distance is measured, which is symbolised by the green bar between two nanoparticle centres in Figure 5C.

If a unified area contains more than two particles, we classify all particles inside of it as agglomerated. In this scenario, our algorithm defines a unified area of all agglomerated particles (‘agglomerated area’), marked by the solid blue lines around the pixel cluster in Figure 5D. The algorithm then continuously checks if the agglomerated area contains unassigned particles. If new particles are detected, they are assigned as agglomerated particles, and the agglomerated area is recalculated. This approach terminates the iterative detection step when no additional particle was detected in the last iteration (Figure 5D).

Figure 5E and F represents the case of complex overlapping origami with many particles. In this case, each particle can belong to a different class. The algorithm checks for each particle class independently on every pixel cluster, and thus can still resolve this issue. In general, agglomerates are determined first (Figure 5E) before the remaining particles are classified (Figure 5F).

All detailed descriptions of the sub‐steps in Figure 4 on an experimental image are provided in the Supplementary Section S3 (Figure S2). For the wide and simple usage of our algorithm, the software package is developed using Python 3.9^18^ in combination with the OpenCV library 4.5.^17^ Our novel developed software package is available to the general public for use or modification (GPLv3).

## 3 RESULTS

We investigated four specimens with different *L*. Detailed information about the strand length of the specimens is given in Table 1.

**FIGURE 6:**
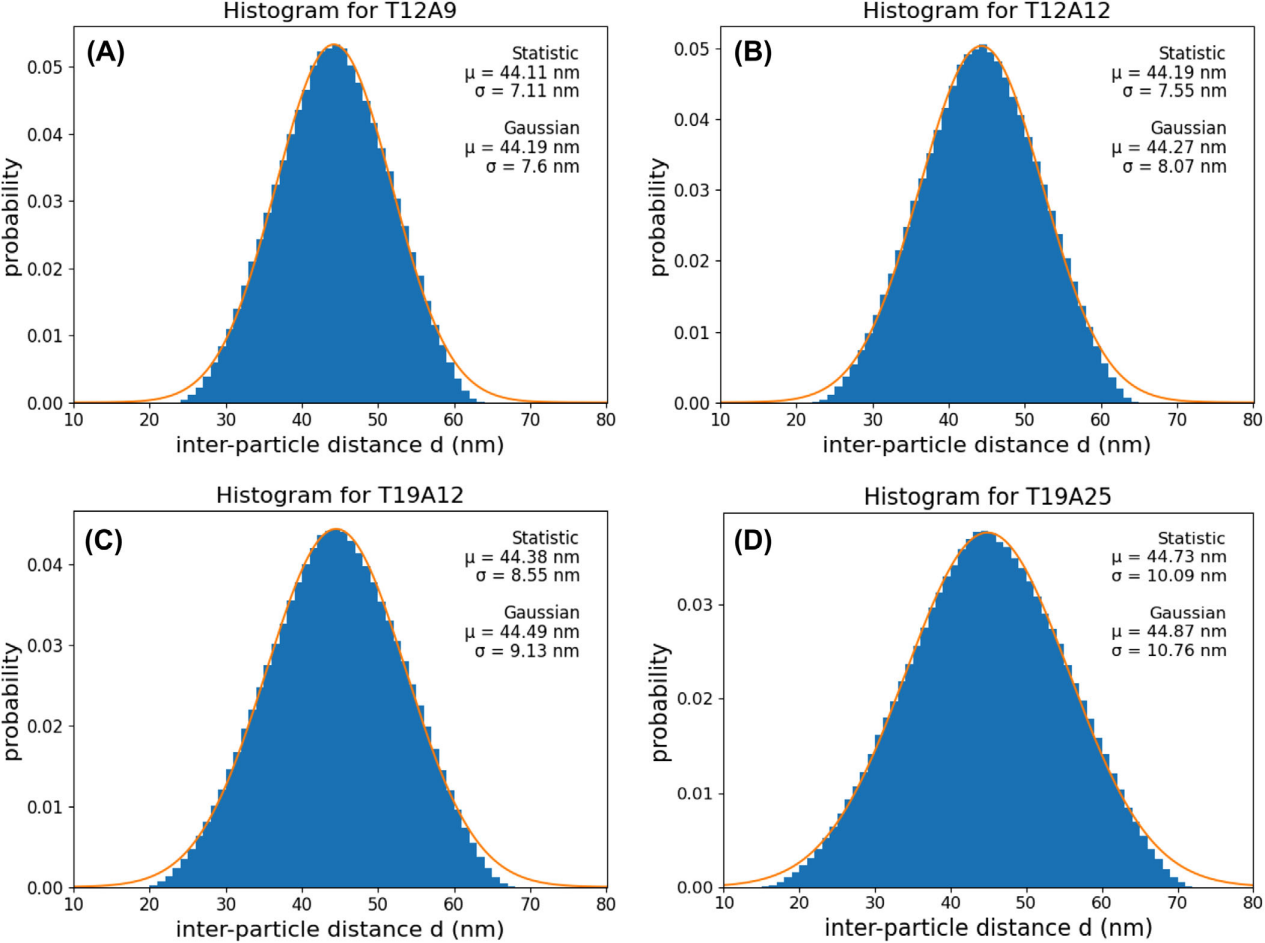
Modelling results for the investigated specimens: (A) T12A9, (B) T12A12, (C) T19A12, and (D) T19A25.

The expected distributions of *d* obtained from our model are shown in Figure 6. The mean value of *d* in each distribution is roughly equal to *d*_0_ with a monomodal distribution, whereas the standard deviation increases with the length of the strand.

**FIGURE 7:**
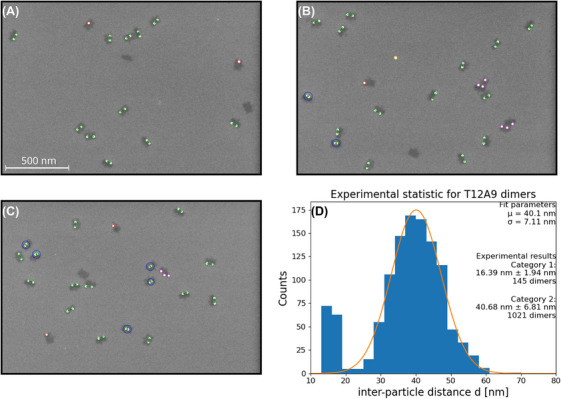
The particle classification of T12A9: (A)–(C) show three exemplary images with different amounts of the category 1 dimer, and (D) interparticle distance statistics after manual removal of ill‐detected dimers.

The measured distribution of *d* for the T12A9 specimen is shown in Figure 7. It is noted that our analysis result shows a bimodal distribution with a minor peak around 17 nm. Since the particle size is roughly 15 nm, the nanoparticles should have physical contact with each other at this distance (blue circles). According to our geometric considerations, the minimum distance between dimers (*d*_min_ = *d*_0_ *–* 2*S’*) should be around 23 nm for this specimen. This minor peak was observed across all specimens (Figure 9). Based on our geometric model, this result is unexpected. Even for the T19A25 specimen, where *d*_min_ overlaps with the minor peak, we only expect a very small share (< 0.1%) of dimers below 20 nm. We therefore assume another mechanism, which is further discussed in the following section.

To estimate the share of the dimers of the minor peak compared to the total amount of dimers, the dimers below the threshold value of *d < d*_min_ were counted. Based on this threshold, dimers were classified into category 1 and category 2 dimers in Figure 7 (D). We used the same approach for the other specimens and determined shares between 10% and 15% of total dimers in each specimen. Despite the existence of minor peaks (category 1 dimers), the distribution of the major peaks (category 2 dimers) fits well with our prediction for *µ* and *σ* based on our model. Therefore, we assume that a dominant tendency of interparticle distance from the formed dimers on the DNA origami can be interpreted based on our model.

However, due to the imperfect model, a direct comparison of standard deviation values between the experiment and model results by simply excluding the minor peak with a certain threshold value is not an ideal approach because the respective *d*_min_ of the longer DNA strands overlaps with the minor peak. Therefore, we instead used the distribution of *d* > 25 nm to fit a Gaussian function for each specimen and compared it with the analogous fitting result from the model.

**FIGURE 8:**
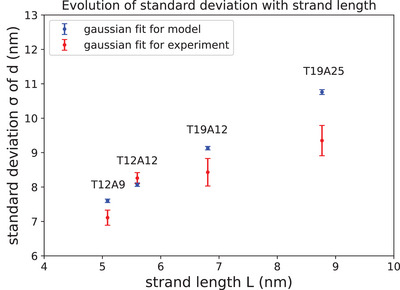
Evolution of *σ* with the *L* (Table 1). The error bars were calculated from the covariance matrix of the parameters of the Gaussian fit.

**FIGURE 9:**
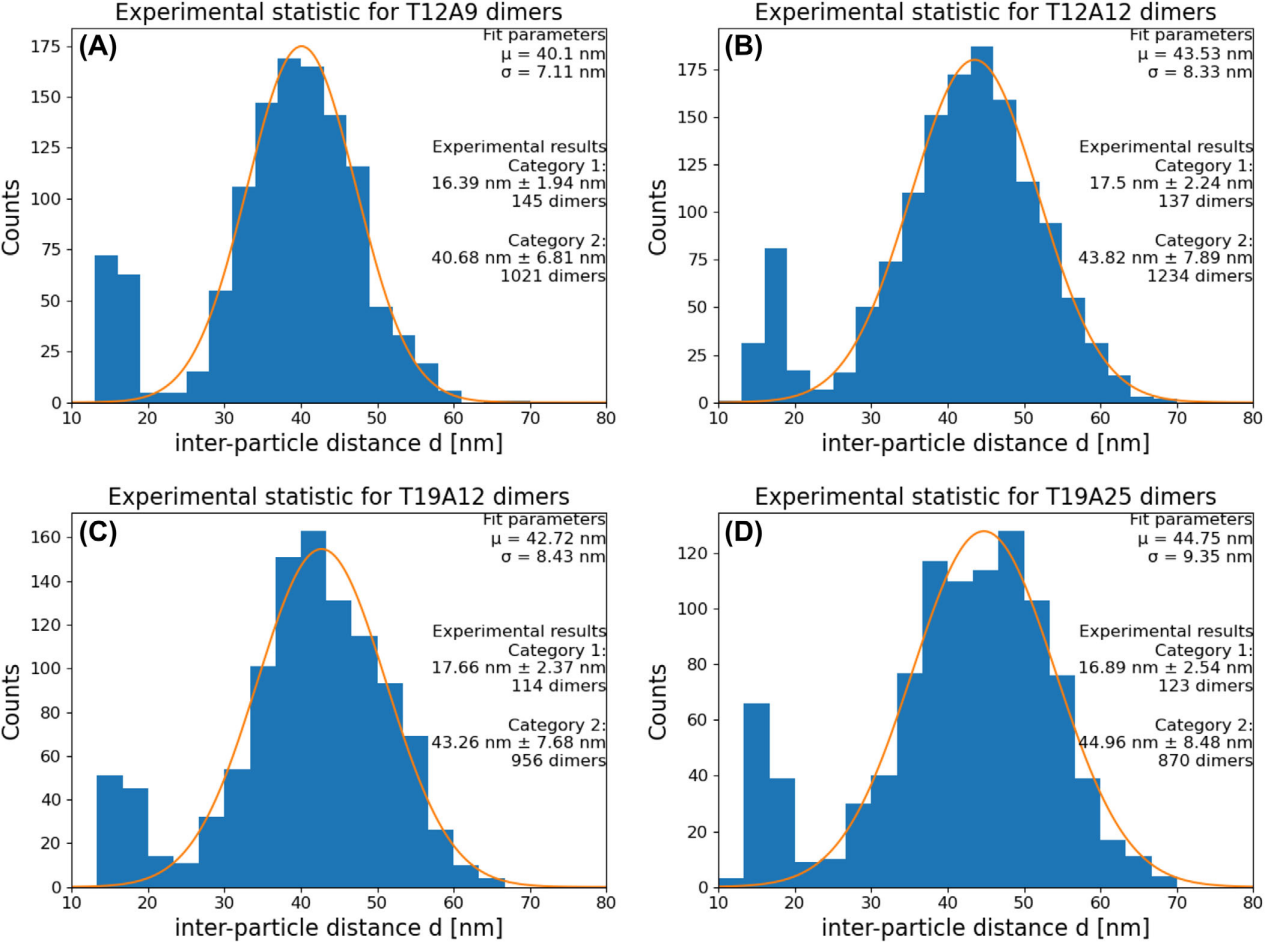
Histograms and Gaussian fit functions for all investigated strand lengths: (A) T12A9, (B) T12A12, (C) T19A12, and (D) T19A25. These histograms had their ill‐detected dimers removed by manual review.

The analyses for the model and the experimental results are shown in Figure 8. The blue points indicate the standard deviations estimated from the model, while the red points represent the standard deviations measured from the experimental data. Upon analysing these results, we observed a continuous increase in *σ* with *L* from distinct specimens (Figure 9). It is evident that this observation shows a good agreement with the tendency of *σ* estimated from our initial hypothesis.

## 4 DISCUSSION

As shown in Figure 8, the predicted trend of *σ* from our model is arguably well reproduced in the experimental results. However, the standard deviation and variations with *L* are generally overestimated in our model. This subtle deviation can be explained by the lack of implementation of the microscopic description for the full DNA binding mechanisms in our simple model. The initial assumption of evenly distributed particle positions within *A*_1, 2_ in Figure 1B can lead to this deviation. Considering our experimental analysis results, an uneven distribution towards the centre of *A*_1, 2_ seems reasonable, leading to a smaller growth rate of *σ* with *L*. Therefore, we suggest that the results from the model should be considered as a simple estimate of the effects of *L* on *σ*. Despite the simplicity of our model, the hypothesis regarding the effects of *L* on *σ* was comparably well reproduced with the experimental results.

Additionally, each distribution contained a second minor peak at smaller distances than what our simple geometric model would allow.

Assuming that drying effects lead to shortening of the anchor point gap, this shortening would be homogeneous in all specimens due to the identical specimen preparation. The geometric overlap requires a *d*_0_, which is smaller than *Δ*_min_ = 2 ∙ (*S'* + *R*). The probability of geometric overlap between the nanoparticles increases if the difference between *Δ*_min_ and *d*_0_ increases. Since *Δ*_min_ increases with *L*, we expect a continuous increase in overlapping dimers. However, the share of category 1 dimers in each specimen does not follow this trend (Figure 9). Hence, the shortening of *d*_0_ due to drying effects can be dismissed as a possible cause for the additional minor peaks.

Alternatively, geometric overlap due to an ill‐estimated *L* from the model can be considered. Since the DNA strand is invisible in SEM micrographs, as shown in Figure 7, their lengths cannot be directly measured. Therefore, we calculated the *L* from the model explained in the Supplementary Section S2. Assuming that the real *L* is larger than the calculated *L* using the model, we would expect a possible geometric overlap due to a *d*_min_. However, under this condition, the expected maximum distance (*d*_max_ = *d*_0_ + 2*S'*) would also increase. Consequently, we would observe a broader distribution of *d*, and the measured *σ* would exceed the model calculations by a significant margin. It is also likely that the slope in Figure 8 would be steeper than the slope predicted by the model calculations. In our study, it is evident that none of these effects occurs. The distributions of *d* tend to be narrower, and the measured *σ* tend to be smaller (Figure 8). We further checked each dataset for dimers above their expected *d*_max_, which varies between 65 and 72 nm, depending on the specimen. The particle classification routines (Figure 5) use *d*_max_ as a parameter for classification. Therefore, we would not be able to detect the dimers above *d*_max_ with our initial analysis. We subsequently repeated the automatic analysis of our data using *d*_max_ = 80 nm. After a manual review, we did not find any dimers exceeding the original values of *d*_max_ in any of our specimens. Thus, our calculation of *L* should be reasonably accurate.

The particle radius (*R)* is the last specimen parameter, which could have a significant effect on the distribution of *d*. According to Equations (1) and (2), an increase in *R* leads to an increase in *S’* and thus the probability of overlapping particles could increase. We measured over 200 nanoparticles from transmission electron microscope images (Figure S3). Despite some variation in the measured *R*, the average radius shows good agreement with the expected value. Additionally, the variations of *R* are too small to account for a significant amount of geometric overlap. Assuming we have the biggest observed particle radius of approximately 9 nm in Equations (1) and (2), we obtain *S’* = 10.83 nm for the specimen T12A9. This is roughly the same as the initial calculation of *S’* for the specimen T12A12, thus geometric overlap for the specimen T12A9 is not expected even in this worst‐case scenario. We can assume that an additional attractive force between nanoparticles can be associated with the minor peak. While Figure 1 shows the poly‐thymine coating with only one outgoing thymine strand, the real coating has multiple outgoing strands.^19^

The free poly‐thymine strands of nanoparticles can interact with each other, thus achieving additional bindings. A finite length between the start and end of the molecule chain, the so‐called persistence length *L*_p_, can be estimated to be roughly 2.2 nm.^20^ Therefore, it seems reasonable that a single‐strand connection between two nanoparticles can shrink to this value and cause touching particles. Our hypothesis supports that the *d* between nanoparticles without this connection would not be affected, producing an independent minor peak next to the major peak without further implications on the distribution of the major peak.

Finally, we briefly discuss the detection reliability of our algorithm. For a large sample size of dimers, the share of correctly detected dimers can be used as a reasonable estimate of the detection reliability. However, even with carefully chosen parameters, poorly detected dimers are occasionally found in manual inspection of individual dimers due to the complexity of the material system. These poor detection results need a manual removal step for the accurate analysis of experimental data. In a binary evaluation (‘correct’ and ‘poor’), the share of poorly detected dimers is complementary to the share of correctly detected dimers. For this reason, we can discuss detection reliability by manually tracking poorly detected dimers. The results of this inspection are summarised in Table 2.

**TABLE 2:** Results of manual dimer inspection for each specimen. Ill‐detected detected dimers were removed from statistical analysis. Furthermore, we recorded the reasons for poor detection and found common trends.

Manually removed dimers per specimen			Reason for dimer removal		
Specimen	Software count	Removed	Ambiguous dimers	Detection error	Etc.
T12A9	1216	50	26	21	3
T12A12	1402	31	25	6	0
T19A12	1282	212	184	10	18
T19A25	1020	27	19	7	1

The vast majority of dimers passed the manual inspection. On average, 3% of the automatically detected dimers were manually removed for the specimens T12A9, T12A12, and T19A25. On the other hand, the specimen T19A12 had a removal rate of 16%. It is noted that severe origami overlaps were evident for this specimen. Therefore, the increased removal rate can be explained by the higher complexity of the dataset. We assume that the specimen preparation procedure for SEM imaging was not optimal. Therefore, the average removal rate for the other 3 specimens can be considered as detection reliability under ideal specimen and imagining conditions.

Additionally, Table 2 lists the reasons for exclusion of the ill‐detected dimers. The majority of excluded dimers were identified as ‘ambiguous dimers’. This group includes all dimers that cannot be unambiguously confirmed in the manual inspection step. It mostly consists of the dimers positioned in the overlapping area of the DNA origami. Since they cannot be confirmed as the dimers on the same origami, they were excluded from the final statistical analysis. It is more common in the specimen T19A12 due to the severe amount of origami overlap and the reason for the higher removal rate in this specimen.

Furthermore, ‘detection errors’ were the second most prevalent reason. This group refers to all classification errors caused by unavoidable CHT detection failure, for example, by missing a particle on a pixel cluster. Despite implementing an effective and robust algorithm, the rare errors in particle detection usually lead to false classification of particles. This error can occur in the case of severely overlapping particles, which can be difficult to correctly detect with the CHT.^21^

Apart from these two reasons, few dimers were removed for other reasons. Due to their overall rarity, we summarised them as a third group called ‘Etc.’.

In addition to its good reliability, our particle classification tool can be easily adapted to other dimer designs due to its modular programming. The code for origami segregation, particle detection, and the detection of different particle classes is designed to operate with minimal overlap among the components. In particular, the particle classification functions (Figure 5) require a list of particle centre positions as input and are not dependant on a particular detection method. Therefore, the tool can be adapted to other particle shapes by substituting CHT for a more appropriate method.

## 5 CONCLUSION

We introduce a novel algorithm for the automatic and reliable analysis of dimers consisting of nanoparticles on the DNA origami in electron microscopy images. Our novel algorithm uses the CHT to detect the nanoparticles on each pixel cluster, while each origami is independently analysed to achieve reliable analysis results. Novel classification routines based on distance and ambiguity criteria categorise each particle into four distinct classes (single particle, monomer, dimer, agglomerate). Because this tool has a modular design, it can be easily expanded and adapted to dimers in other material systems.

We analysed the variation of interparticle distance (*d*) of gold dimers fixed on DNA origami in four different dimer designs with various DNA strand lengths using our algorithm for the secondary electron (SE) images. We achieved control of *d* by positioning nanoparticles on a DNA origami using a combination of poly‐thymine and poly‐adenine single‐stranded DNA. Based on geometric considerations we assumed that the standard deviation (*σ*) in the distribution of *d* is associated with the strand length (*L*) of the DNA strand. For reliable statistical analysis, a large number of dimers is required (∼1000 per specimen). Applying our new algorithm to this complex system enabled us to automatically analyse the required number of dimers in a few minutes.

The preparation method and optimal imaging parameters enabled to distinguish the nanoparticles, the DNA origami, and the silicon wafer with reasonably good contrast. The novel algorithm achieved an overall high accuracy in statistical analysis, providing a high efficiency on dimer detection with only a few errors, which can be easily fixed with manual inspection.

We observed a clear tendency for the growth of *σ* with increasing *L* across all investigated specimens. This tendency can be expected based on our simple model with reasonable geometric considerations. However, an unexpected minor peak in the distribution of *d* was also observed for all specimens. Considering all plausible binding mechanisms previously discussed, we think that this minor peak can be attributed to the additional attractive force between nanoparticles due to the additional DNA strands in the functionalisation.

## CONFLICT OF INTEREST STATEMENT

The authors declare no conflicts of interest.

## Supporting information



Supporting information

## Data Availability

The source code of our tool is available at https://github.com/j‐guckel/automatic‐dimer‐analysis.
