# Supplementary material for: Novel analysis tool for the distance of gold dimers controlled by the DNA strand length on the DNA origami
Source: J Microsc. 2024 Nov 15;297(2):215–26. doi: 10.1111/jmi.13371 (PMC11733842; doi:10.1111/jmi.13371)
Supplement: Supplementary file 1 — Supporting information [file JMI-297-215-s001.docx]

**Supplement**

**S1: Detailed recipe synthesis of modified nanoparticles and DNA origami**

This section explains the detailed steps for the synthesis of the DNA origami and the modification of the gold nanoparticles. The synthesis was identical across all specimens, with the length of the DNA strands being the only change.

***Modification of gold nanoparticles***

We used gold nanoparticles with a diameter of 15 nm from the BBI solutions and mixed them with a BSPP solution (75 µl of H_2_O, 100 mM of BSPP). After several hours of shaking, small amounts of sodium chloride (NaCl) (5 M) were added until the colour changed from red to blue. After this, the particles were removed from liquid by centrifugation (10000 rcf, 30 minutes) and suspended into a mixture of methanol and 2.5 mM BSPP (volume ratio 1:1). This centrifugation-redispersion cycle was repeated one more time. We measured their concentration using UV-Vis spectroscopy before continuing further. The solution was mixed with single-stranded poly-thymine DNA modified with thiol groups in a 1×TAE buffer. The molar concentration of poly-thymine DNA was 300 times higher than the molar concentration of the nanoparticles. We added NaCl (5 M) to the solution in 4 steps. After each step, the particles were sonicated for 10 seconds and shaken for 10 minutes. The final concentration of NaCl in the solution was around 500 mM. Afterwards, the mixture is shaken for several hours, and the particles are filtered out by a final centrifugation step (10000 rcf).

***Modification of DNA origami***

The DNA origami was designed with CaDNAno. Each origami is a rectangular structure made of 24 double-stranded DNA on an M13mp18 scaffold connected by 188 staples. Six staples (3 at each anchor point) were extended with poly-adenine single-stranded DNA. The origami was synthesised by mixing scaffold (1 pM) and staples (20 pM) in a folding buffer. The folding buffer is a 1×TAE buffer with 12.5 mM MgCl_2_ solved into it. The mixture was heated up to 90 °C before being cooled down to room temperature (cooling rate 1 °C/min). The DNA origami were then purified multiple times by centrifugation (10000 rcf) and washed in a folding buffer after each step. The concentration of DNA origami was then determined by UV-vis spectroscopy.

**S2: Calculation of the strand length**

We calculated the *L* by using the model shown in [Figure S1](#S1). One end piece of the poly-thymine strand is connected to the nanoparticle through thiol modification, whereas one end piece of the poly-adenine strand is connected to the DNA origami.

We assume that the loose ends of both strands connect via hydrogen bonding, thus forming double-stranded DNA with overlapping base pairs. The entire strand therefore consists of 3 sub-strands with single-stranded poly-thymine near the nanoparticle, single strand poly-adenine near the origami and double-stranded DNA in-between.

However, the exact number of double-stranded base pairs is unknown. We assume 9 base pairs as minimum requirement for stability of the double-strand, but more base pairs are still considered possible. Therefore, each configuration would result in a different *L*. For this reason, we estimate *L* using the unweighted mean value of all possible configurations.

In general, we assume the length of each sub-strand is proportional to its number of base pairs. We use 0.676 nm per base pair for single-stranded DNA^20^ and 0.34 nm per base pair for double-stranded DNA^22^. However, this only applies if the length of a sub-strand is smaller than its persistence length *L_p_*. According to IUPAC^23^, the persistence length is defined as the end-to-end projection of a worm-like chain of infinite chain length, e.g. a chain of identical amino acid sequences. Therefore, our model does not allow for any sub-strand to surpass its *L_p_*. Double-stranded DNA has a persistence length of 50 nm (147 base pairs), while the maximum length of double-stranded DNA in our experiment is 19 base pairs. Therefore, the length of double-stranded DNA is not affected by *L_p_*. On the other hand, the persistence length of single-stranded DNA is much shorter with *L_p_* = 2.223 nm (≈ 3.3 base pairs)^20^. Therefore, any single-stranded DNA segment above 3 base pairs is capped by *L_p_*.

**S3: Additional experimental data**

**List of Supplementary Figures and Tables:**


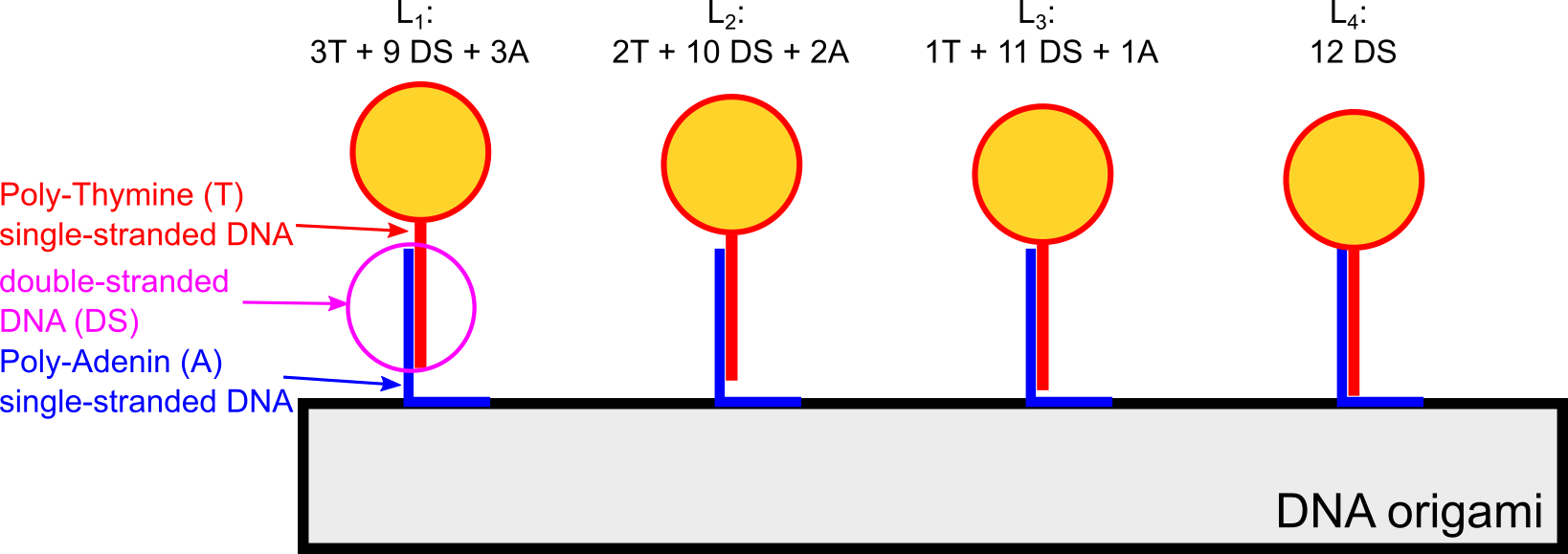


**F I G U R E S 1** Every possible strand configuration for the specimen T12A12. The strand length is calculated as the mean of all configurations.


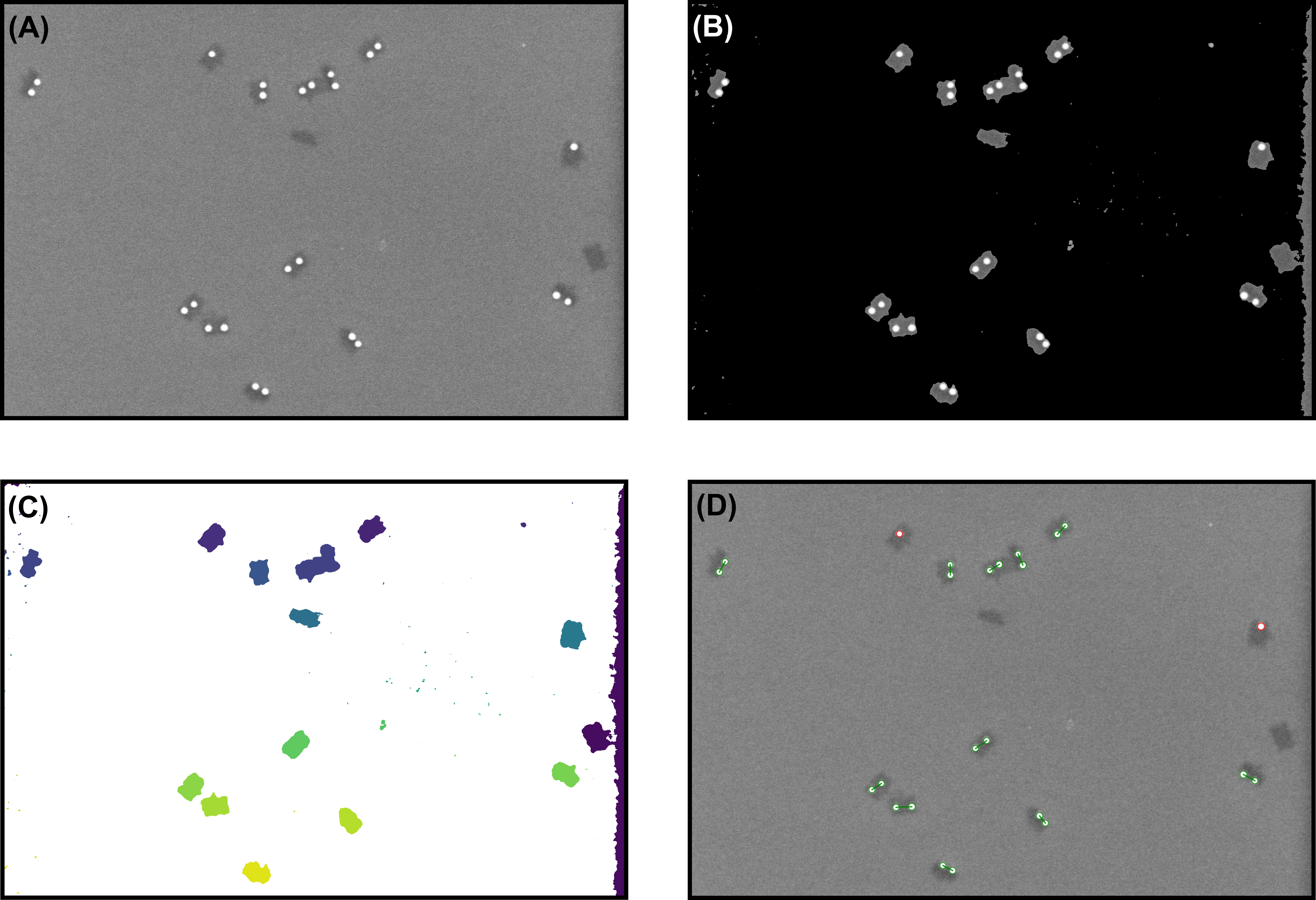


**F I G U R E S 2** Exemplary experimental image of the dimer particle classification routine: (A) original image, (B) image after thresholding, (C) image after labelling, and (D) classification overlay.


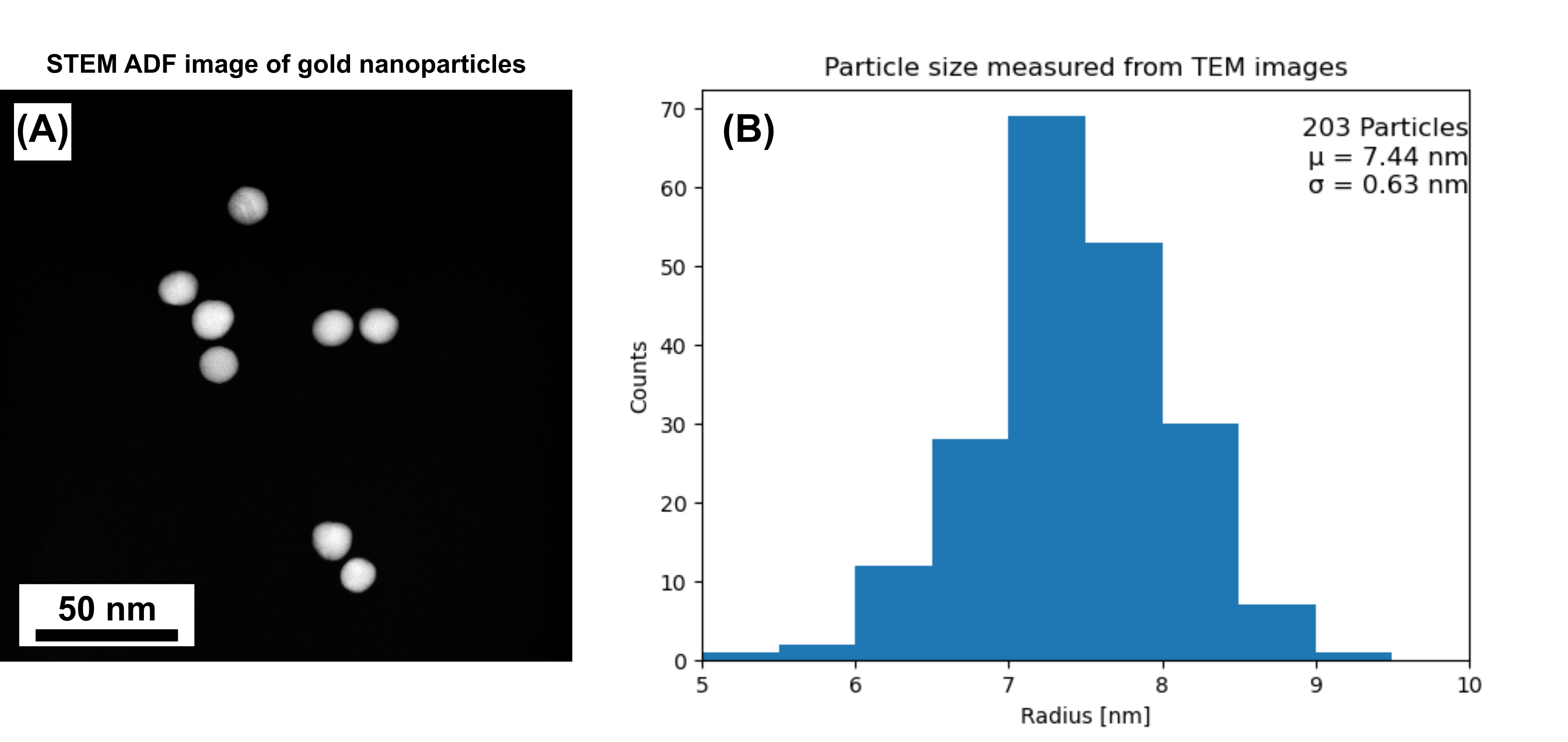


**F I G U R E S 3** Size measurement of gold nanoparticles with TEM (A) STEM-ADF image, and (B) particle radius histogram. The particle radius was obtained with CHT.
